# 18β-Glycyrrhetinic Acid Exerts Subgroup-Specific Antitumor Activity and Enhances Radiosensitivity in Medulloblastoma

**DOI:** 10.3390/cancers18193233

**Published:** 2026-10-08

**Authors:** Francesca Palone, Luca Marchetti, Marco Corso, Emiliano Fratini, Ilaria De Stefano, Ilaria Di Sarcina, Simonetta Pazzaglia, Mariateresa Mancuso

**Affiliations:** 1 Biotechnologies Division, Department for Sustainability, Italian National Agency for New Technologies, Energy and Sustainable Economic Development (ENEA), 00123 Rome, Italy; luca.march46@gmail.com (L.M.); marco.corso2001@gmail.com (M.C.); emiliano.fratini@enea.it (E.F.); ilaria.destefano@enea.it (I.D.S.); simonetta.pazzaglia@enea.it (S.P.); 2 Radiation Systems and Application Division, Nuclear Department, Italian National Agency for New Technologies, Energy and Sustainable Economic Development (ENEA), 00123 Rome, Italy; ilaria.disarcina@enea.it

**Keywords:** medulloblastoma, 18β-glycyrrhetinic acid, radiosensitization, radiotherapy, SHH signaling, AMPK/NF-κB, natural compounds

## Abstract

Background: Radiotherapy (RT) is a cornerstone of medulloblastoma (MB) treatment, but its efficacy may be limited by intrinsic or acquired radioresistance, while radiation-related late effects remain a major concern in pediatric patients. 18β-Glycyrrhetinic acid (18β-GA), a bioactive triterpenoid derived from *Glycyrrhiza glabra*, has shown antitumor activity in several malignancies. This study evaluated its antitumor and radiosensitizing effects in biologically distinct MB models. Methods: DAOY cells, used as an SHH-associated, TP53-mutant model, and D283 cells, commonly considered a Group 3/Group 4-associated model, were treated with 18β-GA (2.5–5 μM), γ-radiation (2–4 Gy), or their combination. Cell viability, clonogenic survival and migration were assessed. Apoptosis was evaluated by caspase-3/7 activity and cleaved caspase-3 expression. Modulation of AMPK/NF-κB and SHH signaling was examined by immunoblotting and RT-qPCR. Results: 18β-GA reduced cell viability in a dose- and time-dependent manner and significantly reduced wound closure in DAOY cells and migratory capacity in D283 cells. D283 cells showed greater apoptotic sensitivity, accompanied by increased total and phosphorylated AMPK and reduced NF-κB p65 expression. In DAOY cells, 18β-GA reduced the expression of SHH-associated transcriptional markers, including GLI1, SOX2, CCND1, and MYC. Most importantly, 18β-GA significantly enhanced radiation response in both cell lines, producing greater reductions in viability and clonogenic survival than irradiation alone (*p* < 0.01). Combined treatment with 5 μM 18β-GA and 4 Gy reduced the surviving fraction to 0.013 in DAOY cells and approximately 0.01 in D283 cells (*p* < 0.001 versus radiation alone). This radiosensitizing effect was accompanied by increased caspase-3/7 activity and cleaved caspase-3 expression. Conclusions: 18β-GA exerts antitumor activity in biologically distinct MB models through differential modulation of SHH and AMPK/NF-κB signaling, while converging on enhanced apoptotic susceptibility and a profound loss of clonogenic survival after irradiation. These findings provide a strong rationale for further preclinical evaluation of 18β-GA as a potential radiosensitizing adjuvant in MB.

## Simple Summary

Medulloblastoma is the most common malignant brain tumor in children, and radiotherapy is an essential part of treatment. However, some tumor cells can be resistant to radiation, reducing treatment effectiveness and contributing to disease recurrence. In this study, we investigated the effects of 18β-glycyrrhetinic acid, a natural compound derived from licorice, in two biologically distinct medulloblastoma cell models. We found that this compound reduced tumor cell growth and cell motility and increased the sensitivity of cancer cells to radiation. We also identified differences in the molecular mechanisms involved in the two tumor subgroups, suggesting that biological characteristics may influence treatment response. These findings improve our understanding of how natural compounds can modulate radiation response and support further preclinical studies to evaluate their potential role in medulloblastoma treatment.

## 1. Introduction

MB is a malignant tumor of the central nervous system (CNS) and represents the most common malignant brain tumor in children, although it is relatively rare in adults [1,2]. Peak incidence occurs between 5 and 6 years of age, with a male-to-female ratio of approximately 2:1 [3]. Despite advances in multimodal therapy, including maximal safe surgical resection, craniospinal irradiation (CSI), and multi-agent chemotherapy, a substantial proportion of patients continue to experience disease recurrence or long-term treatment-related morbidity [4,5]. While five-year survival rates exceed 80% in standard-risk patients, outcomes remain less favourable in high-risk cases, including those presenting with metastatic disease, residual tumor burden, or MYC amplification, for whom survival may fall below 70% [6,7]. Furthermore, the administration of CSI in very young children is constrained by the risk of severe long-term toxicities, including neurocognitive impairment, endocrinopathies, ototoxicity, and cerebrovascular complications [8].

MB is now recognised as a biologically heterogeneous disease comprising four major molecular subgroups: WNT-activated, SHH-activated, Group 3 and Group 4, each displaying distinct genetic, epigenetic, clinical, and prognostic characteristics [9]. WNT-activated MB is associated with excellent survival outcomes, whereas Group 3 tumors, particularly those harbouring MYC amplification, exhibit the poorest prognosis [10]. The integration of molecular classification into clinical practice has enabled the development of risk-adapted therapeutic approaches, including treatment de-escalation in favourable-risk patients and the investigation of targeted therapies. Nevertheless, treatment resistance and radiation-associated toxicity remain major obstacles to further improving clinical outcomes, highlighting the need for novel therapeutic strategies capable of enhancing tumor control while minimising adverse effects on the developing brain. Radiosensitization has emerged as an attractive strategy to address this challenge. Agents capable of increasing tumor cell susceptibility to ionising radiation (IR) may improve the therapeutic ratio by enhancing local tumor control at conventional radiation doses or by enabling radiation dose reduction without compromising efficacy [11]. In recent years, naturally derived compounds have gained increasing attention as potential radiosensitizers due to their favourable safety profiles and ability to modulate multiple cellular pathways involved in proliferation, survival, inflammation, oxidative stress, and apoptosis [12,13]. Among these compounds, 18β-glycyrrhetinic acid (18β-GA), a bioactive pentacyclic triterpenoid derived from *Glycyrrhiza glabra* (licorice), has demonstrated promising anti-cancer activity in a variety of tumor models [14]. Previous studies have shown that 18β-GA inhibits tumor growth through multiple mechanisms, including induction of apoptosis and suppression of proliferation, migration, and invasion, mediated by modulation of signaling pathways such as ROS/MAPK/STAT3/NF-κB and EGFR/ERK [15,16,17]. Beyond its direct anti-neoplastic effects, increasing evidence indicates that 18β-GA modulates cellular responses to oxidative stress and radiation-induced damage through activation of cytoprotective pathways, including the Nrf2/HO-1 axis, and through the regulation of ROS-mediated signaling networks [18,19,20]. Moreover, previous investigations suggest that glycyrrhizin-derived compounds exhibit a favourable safety profile at biologically active concentrations, further supporting their evaluation as potential adjuncts to conventional anticancer therapies. Despite these promising observations, the radiosensitizing potential of 18β-GA in MB remains largely unexplored. This issue is particularly relevant given the complex mechanisms underlying radioresistance in MB, including activation of pro-survival pathways, persistence of stem-like tumor cell populations, adaptive stress responses, and subgroup-specific molecular alterations [21,22]. Considering the marked biological heterogeneity of MB, different molecular subgroups may also exhibit distinct sensitivities to 18β-GA and radiation-based combination therapies.

In the present study, we investigated the anti-tumor and radiosensitizing effects of 18β-GA in human MB cell models representative of biologically distinct disease subgroups, namely SHH-activated/TP53-mutant DAOY cells and stem-like Group 3/4 D283 cells. We evaluated the impact of 18β-GA alone and in combination with ionising radiation on cell viability, clonogenic survival, migration, apoptosis, and key molecular pathways associated with tumor progression and radiation response, including SHH and AMPK/NF-κB signaling. By characterising subgroup-specific responses to treatment, we sought to provide preliminary evidence supporting the development of 18β-GA as a novel radiosensitizing agent for MB and to explore its potential application in molecularly stratified therapeutic approaches.

## 2. Materials and Methods

### 2.1. Cell Lines

The human MB cell lines DAOY (ATCC HTB-186; American Type Culture Collection, Manassas, VA, USA; RRID:CVCL_1167) and D283 Med (ATCC HTB-185; RRID:CVCL_1155) were obtained from the American Type Culture Collection (ATCC, Manassas, VA, USA). DAOY cells, representative of the SHH subgroup, and D283 Med cells, commonly used as a Group 3/Group 4-like model, were cultured in Minimum Essential Medium supplemented with 10% fetal bovine serum and 1% penicillin–streptomycin under standard conditions (37 °C, 5% CO_2_). Cells were routinely tested for mycoplasma contamination.

### 2.2. Reagents

18β-GA (Sigma-Aldrich, St. Louis, MO, USA) was dissolved in DMSO to prepare a 40 mM stock solution and diluted in culture medium to final concentrations of 2.5–40 µM.

Dorsomorphin (Compound C; Cell Signaling Technology, Danvers, MA, USA) was used as an AMPK inhibitor at a final concentration of 10 µM.

Vehicle-treated controls received equivalent concentrations of DMSO (0.1% *v*/*v*).

### 2.3. Combined Treatment with 18β-GA and γ-Radiation

DAOY and D283 cells at 80–90% confluence were pre-treated with 18β-GA (2.5 or 5 µM) for 24 h prior to irradiation. Cells were subsequently exposed to γ-radiation at the Calliope facility (ENEA Casaccia Research Centre, Rome, Italy), a pool-type γ-irradiator equipped with a 60Co source operating at an average photon energy of 1.25 MeV. The irradiation system consists of a planar arrangement of 25 60 Co rods with an active area of 41 cm × 75 cm housed within a high-volume shielded irradiation cell (7.0 m × 6.0 m × 3.9 m) [23,24]. Cells were irradiated in T75 culture flasks positioned horizontally at a dose rate of 23.83 Gy/h, receiving total absorbed doses of 2 Gy or 4 Gy. All dosimetric values were referred to water. Immediately after irradiation, cells were returned to standard culture conditions (37 °C, 5% CO_2_) and maintained for 24 h before further analyses. For clonogenic assays, irradiated cells were harvested and re-seeded at the indicated densities for colony formation.

### 2.4. Cell Viability Assay

Cell viability was evaluated by seeding 5 × 10^3^ DAOY cells and 1 × 10^4^ D283 cells per well in white 96-well plates (Cat. No. 3610, Corning, NY, USA) containing 200 µL of complete culture medium. Cells were treated with increasing concentrations of 18β-GA (2.5–40 µM).

Cell viability was monitored using the RealTime-Glo™ MT Cell Viability Assay (Promega, Madison, WI, USA) according to the manufacturer’s instructions. Luminescence was measured as Relative Luminescence Units (RLU) using a GloMax^®^ Discover Microplate Reader (Promega) at 0, 24, 48, and 72 h after treatment. Results were expressed as the percentage of viable cells relative to the corresponding vehicle-treated control, which was defined as 100%. All experiments were performed in triplicate and repeated independently at least three times.

### 2.5. Clonogenic Assay

Clonogenic survival was assessed to evaluate the long-term reproductive capacity of MB cells following treatment, an established measure of radiosensitivity. DAOY and D283 cells were pre-treated with 18β-GA for 24 h and subsequently exposed to γ-radiation (2 and 4 Gy). Following treatment, DAOY (200 cells/well) and D283 (500 cells/well) cells were seeded in poly-D-lysine-coated 6-well plates and maintained under standard culture conditions to allow colony formation.

After 8 days (DAOY) or 14 days (D283), colonies were fixed with methanol and stained with 0.5% crystal violet in 50% methanol. Colonies containing at least 50 cells were scored as survivors and counted manually.

The plating efficiency (PE) was calculated as the ratio of colonies formed to cells seeded in the corresponding non-irradiated control group. The surviving fraction (SF) was calculated by normalizing the colony-forming ability of treated cells to the plating efficiency of vehicle-treated controls. For combination-treatment groups SF were normalized to the corresponding non-irradiated control at each 18β-GA concentration to account for the intrinsic anti-clonogenic effect of the compound. Specifically, the normalized SF for each irradiated condition was calculated by dividing the surviving fraction at a given radiation dose by the surviving fraction of the corresponding sham-irradiated group treated with the same 18β-GA concentration. Direct statistical comparisons were performed between radiation-alone and combined-treatment groups at each radiation dose. The normalized SF, corresponding statistical analyses (*p*-values), and DEF_50_ estimates are reported in Supplementary Table S2. Dose enhancement factors at 50% survival (DEF_50_) were estimated by fitting survival data to the linear-quadratic model. DEF_50_ was calculated as the ratio between the radiation dose required to achieve a surviving fraction of 0.50 in untreated cells and that required in 18β-GA-treated cells. In DAOY cells, DEF_50_ was not considered reliably estimable because the survival curves intersected within the experimentally investigated dose range.

### 2.6. Migration Assay

#### 2.6.1. Wound-Healing Assay

Migration of DAOY cells was evaluated using a wound-healing assay. Confluent cell monolayers grown in 6-well plates were scratched with a sterile pipette tip and treated with 18β-GA, γ-radiation, or the combination treatment. To minimize the contribution of cell proliferation to wound closure, cells were maintained in serum-free medium throughout the assay. Images were acquired at 0, 24, 48, and 72 h, and wound closure was quantified using ImageJ software version 1.52 (National Institutes of Health, Bethesda, MD, USA) and expressed as the percentage of closure relative to the initial wound width.

#### 2.6.2. Transwell Migration Assay

Migration of D283 cells was assessed using Transwell inserts with 8 µm pore membranes (Millipore, Bedford, MA, USA). Serum-starved cells (1 × 10^5^) were seeded in the upper chamber in serum-free medium, whereas the lower chamber contained complete medium supplemented with 10% fetal bovine serum as a chemoattractant. After 24 h, migrated cells were fixed, stained with crystal violet, and quantified in five randomly selected microscopic fields per insert. Results were expressed as the mean number of migrated cells per field relative to vehicle-treated controls. All experiments were performed in triplicate and repeated independently at least three times.

### 2.7. Apoptosis Assay

Apoptosis was assessed by measuring caspase-3/7 activity using the Caspase-Glo^®^ 3/7 Assay (Promega) according to the manufacturer’s instructions. DAOY and D283 cells were seeded in white 96-well plates, treated as indicated, and analyzed 24 h later. Luminescence was measured using a GloMax^®^ Discover Microplate Reader (Promega) and expressed as relative luminescence units (RLU) normalized to vehicle-treated controls. All experiments were performed in triplicate and repeated independently at least three times.

### 2.8. Gene Expression Analysis

Total RNA was extracted using the RNeasy Mini Kit (QIAGEN, Hilden, Germany) and reverse-transcribed using the iScript™ cDNA Synthesis Kit (Bio-Rad, Hercules, CA, USA). Quantitative real-time PCR (RT-qPCR) was performed using SYBR Green chemistry on a CFX96 Touch Real-Time PCR Detection System (Bio-Rad). Expression levels of *GLI1*, *SOX2*, *BCL2*, *CCND1*, and *MYC* were normalized to GAPDH and calculated using the 2−ΔΔCt method. Primer sequences are provided in Supplementary Table S1. All reactions were performed in technical triplicate and repeated in at least three independent biological experiments.

### 2.9. Immunoblotting

Cells were lysed in ice-cold lysis buffer containing protease and phosphatase inhibitors. Equal amounts of protein (30 µg) were separated by SDS–PAGE and transferred onto PVDF membranes. Membranes were incubated with primary antibodies against AMPK, phosphorylated AMPK (p-AMPK), NF-κB p65, cleaved caspase-3, and β-actin (Cell Signaling Technology, Danvers, MA, USA; Sigma-Aldrich, St. Louis, MO, USA). Following washing with TBS-T, membranes were incubated with HRP-conjugated secondary antibodies. Immunoreactive bands were detected by enhanced chemiluminescence (ECL) and quantified by densitometric analysis. Protein expression levels were normalized to β-actin. Representative immunoblots are derived from at least three independent experiments. The original uncropped Western blot images are provided in Supplementary Figure S3.

### 2.10. Statistical Analysis

Data are presented as mean ± standard deviation (SD) from at least three independent experiments (*n* = 3). Statistical analyses were performed using GraphPad Prism version 8.0.2 (GraphPad Software, San Diego, CA, USA).

Comparisons between two groups were performed using an unpaired Student’s *t*-test, with Welch’s correction applied when appropriate. Comparisons among multiple groups were performed using one-way or two-way analysis of variance (ANOVA) followed by Tukey’s multiple-comparison post hoc test.

Differences were considered statistically significant at *p* < 0.05. Statistical significance was indicated as follows: * *p* < 0.05, ** *p* < 0.01, *** *p* < 0.001, and **** *p* < 0.0001.

## 3. Results

### 3.1. 18β-GA Exerts Anti-Tumor Effects in MBCells

**Figure 1:**
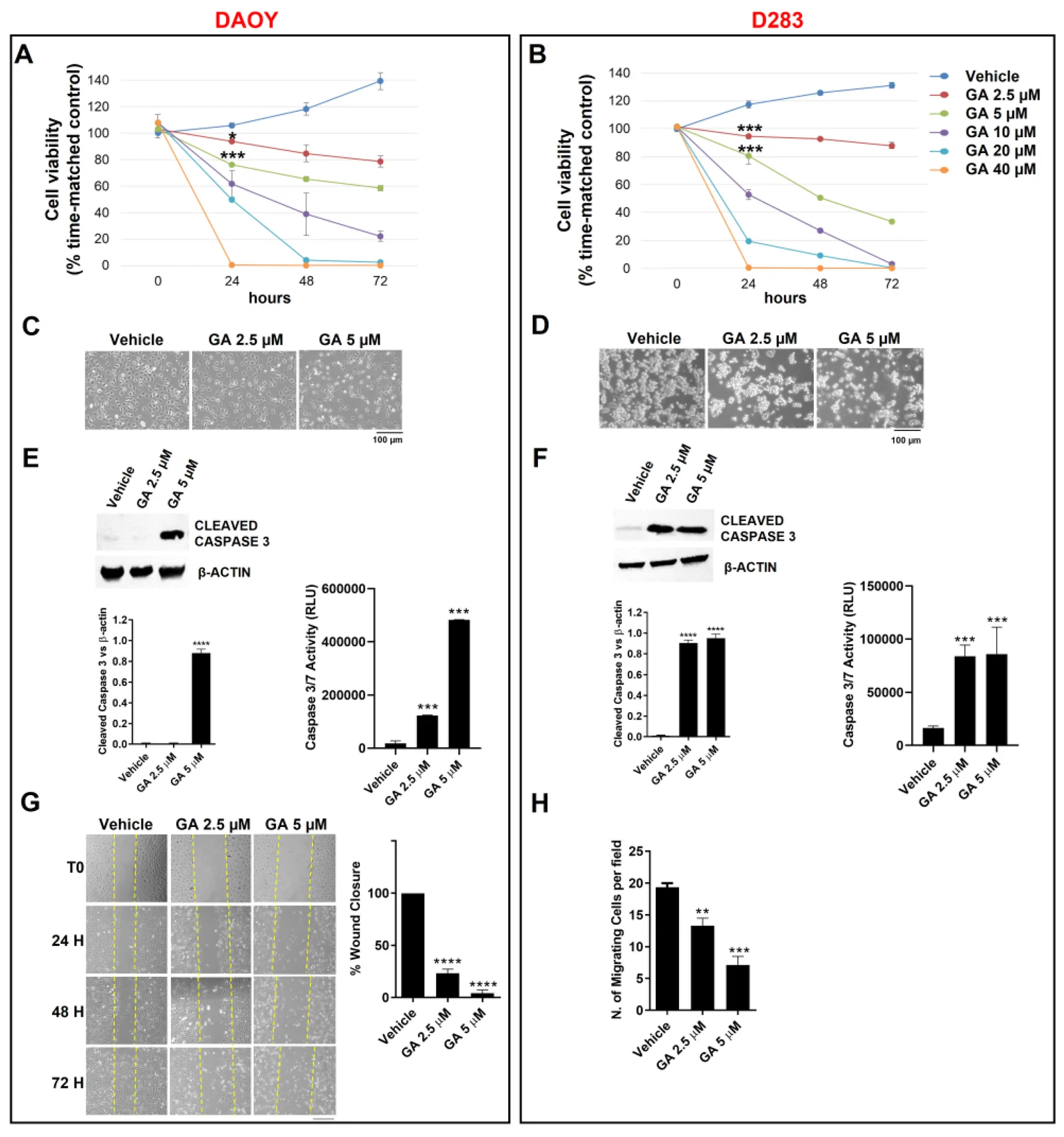
18β-GA exerts anti-tumor effects in MB cells. DAOY and D283 cells were treated with increasing concentrations (2.5–40 μM) of 18β-GA. Cell viability was evaluated using the RealTime-Glo™ MT Cell Viability Assay and expressed as the percentage of viable cells relative to time-matched vehicle-treated controls after 24, 48, and 72 h of treatment (**A**,**B**). Representative phase-contrast images (10× magnification; scale bar = 100 µm) showing reduced cell density and morphological alterations following treatment with vehicle, 2.5 µM, and 5 µM 18β-GA for 24 h are shown (**C**,**D**). Apoptosis was assessed after 24 h of treatment by evaluating cleaved caspase-3 expression by immunoblotting, with corresponding densitometric analyses normalized to β-actin, and caspase-3/7 activity (**E**,**F**). The original uncropped Western blot images are provided in Supplementary Figure S3. The effect of 18β-GA on wound closure was assessed using a wound-healing assay in DAOY cells (10× magnification; scale bar = 100 µm), where dashed lines indicate the initial wound boundaries immediately after scratching (0 h), and a Transwell migration assay in D283 cells, with corresponding quantitative analyses shown in panels (**G**) and (**H**), respectively. Data are presented as mean ± SD from three independent experiments (*n* = 3). Statistical significance was determined using an unpaired Student’s *t*-test with Welch’s correction (**A**,**B**) or one-way ANOVA followed by Tukey’s multiple-comparison post hoc test (**E**–**H**), as appropriate. * *p* < 0.05; ** *p* < 0.01; *** *p* < 0.001; **** *p* < 0.0001.

To evaluate the anti-tumor activity of 18β-GA, DAOY and D283 MB cells were treated with increasing concentrations of the compound (2.5–40 µM) for 24, 48, and 72 h. Cell viability analysis revealed a significant dose- and time-dependent reduction in both cell lines (Figure 1A,B). Dose–response curves suggested increasing sensitivity over time, with approximately 5 µM reducing cell viability by about 50% at 72 h in DAOY cells and 48 h in D283 cells, indicating increased sensitivity to prolonged exposure. Based on these findings, tested concentrations of 18β-GA (2.5 and 5 µM for 24 h) were selected for subsequent mechanistic and radiosensitization experiments. Under these conditions, viability was reduced by 6.4 ± 0.9% and 23.1 ± 0.4% in DAOY cells and by 5.4 ± 1.5% and 19.6 ± 0.6% in D283 cells following treatment with 2.5 and 5 µM 18β-GA, respectively.

Phase-contrast microscopy revealed a concentration-dependent decrease in cell density in both cell lines, accompanied by marked morphological changes, including cell rounding, shrinkage, and reduced adherence compared with vehicle-treated controls (Figure 1C,D).

To determine whether the reduction in cell viability was associated with apoptotic cell death, cleaved caspase-3 expression and caspase-3/7 activity were evaluated following treatment. In DAOY cells, a significant increase in cleaved caspase-3 expression was observed only after exposure to 5 µM 18β-GA, whereas caspase-3/7 activity was significantly increased at both concentrations, with higher levels observed at 5 µM (Figure 1E). In contrast, D283 cells displayed greater sensitivity to 18β-GA, showing a significant increase in both cleaved caspase-3 expression and caspase-3/7 activity already at 2.5 µM (Figure 1F). These findings suggest that D283 cells are more susceptible than DAOY cells to 18β-GA-induced apoptosis.

To investigate the effect of 18β-GA on the migratory capacity of MB cells, migration was evaluated using a wound-healing assay in DAOY cells and a Transwell migration assay in D283 cells. In DAOY cells, treatment with 18β-GA significantly impaired wound closure in a dose-dependent manner (Figure 1G). After 72 h, wound closure was reduced to 23.1 ± 4.0% and 4.0 ± 3.0% following treatment with 2.5 µM and 5 µM 18β-GA, respectively, corresponding to approximately 70% and 90% inhibition relative to vehicle-treated controls. Similarly, Transwell migration assays revealed a marked reduction in the migratory capacity of D283 cells (Figure 1H). The number of migrated cells decreased from 19.3 ± 2.1 cells/field in control cultures to 13.3 ± 1.2 and 7.1 ± 1.3 cells/field following treatment with 2.5 µM and 5 µM 18β-GA, respectively, corresponding to approximately 30% and 65% inhibition of migratory capacity.

Overall, these findings indicate that 18β-GA suppresses MB cell growth and migration while promoting apoptotic cell death, supporting its potential as a therapeutic agent against MB.

### 3.2. AMPK/NF-κB Signaling Contributes to the Effects of 18β-GA in D283 Cells

**Figure 2:**
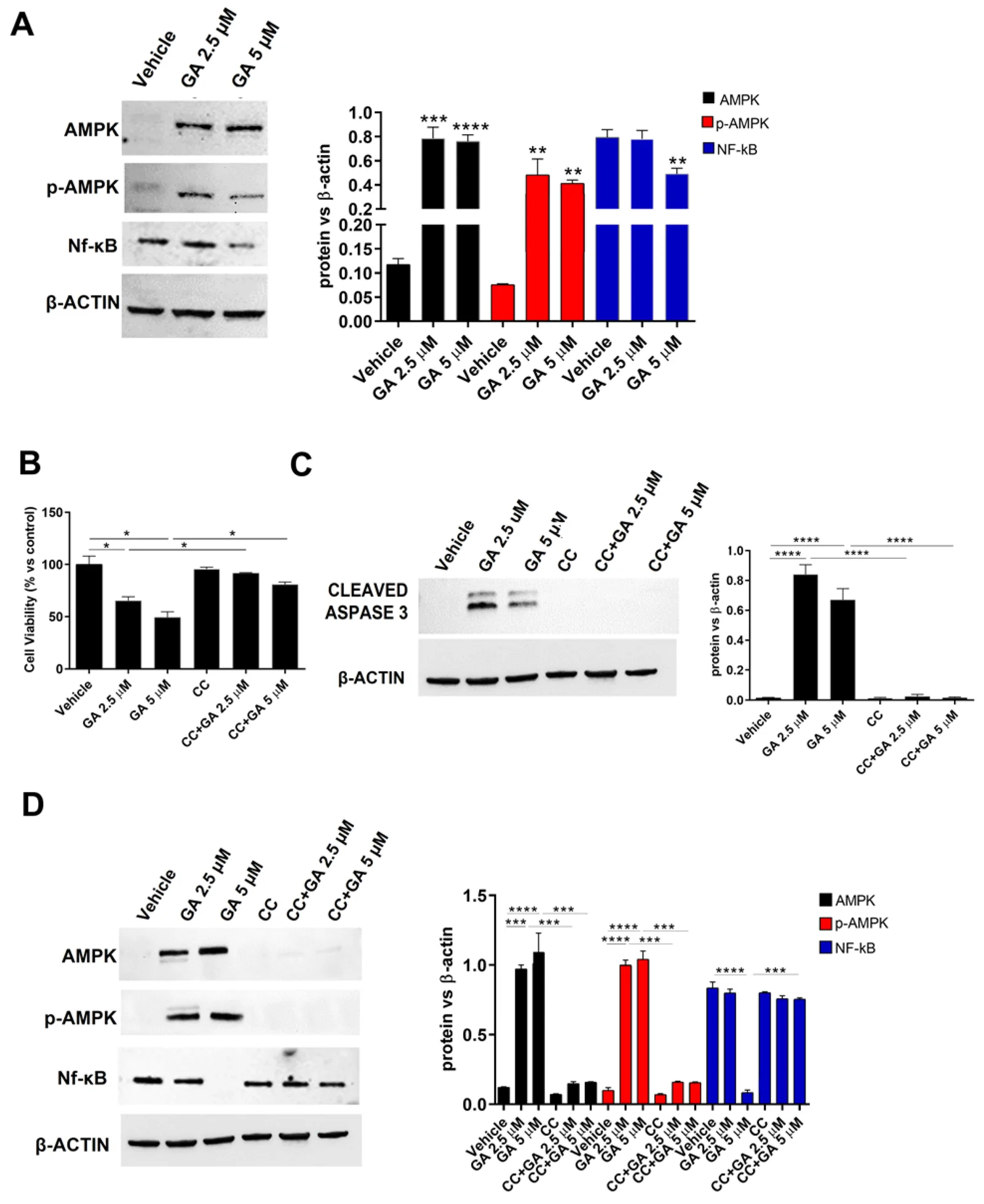
AMPK/NF-κB signaling contributes to the effects of 18β-GA in D283 cells. D283 cells were treated with 18β-GA (2.5 and 5 µM) for 24 h. Representative immunoblots and corresponding densitometric analyses of total AMPK, phosphorylated AMPK (p-AMPK), and NF-κB p65 show increased AMPK expression and phosphorylation together with reduced NF-κB p65 protein levels following treatment (**A**). Cell viability analysis performed in D283 cells pre-treated with the AMPK inhibitor Compound C (10 µM) prior to 18β-GA exposure demonstrates restoration of cell viability compared with cells treated with 18β-GA alone (**B**). Representative immunoblots and densitometric analysis of cleaved caspase-3 expression indicate attenuation of 18β-GA-induced apoptotic signaling following AMPK inhibition (**C**). Immunoblot analysis further demonstrates that Compound C suppresses 18β-GA-induced AMPK phosphorylation and restores NF-κB p65 expression levels (**D**). The original uncropped Western blot images are provided in Supplementary Figure S3. Data are presented as mean ± SD from three independent experiments (*n* = 3). Statistical significance was determined using one-way ANOVA followed by Tukey’s multiple-comparison post hoc test. For each treatment condition, comparisons were made against the corresponding vehicle-treated control (0 μM 18β-GA). * *p* < 0.05; ** *p* < 0.01; *** *p* < 0.001; **** *p* < 0.0001.

To investigate the molecular mechanisms underlying the anti-tumor activity of 18β-GA, the involvement of the AMPK/NF-κB signaling axis was evaluated. In DAOY cells, 18β-GA treatment did not significantly affect AMPK expression, phosphorylation, or NF-κB p65 expression, suggesting that this pathway is not a major mediator of the cellular response to treatment (Supplementary Figure S1). In contrast, D283 cells displayed a marked molecular response to 18β-GA. Immunoblot analysis revealed increased AMPK expression and phosphorylation, accompanied by reduced NF-κB p65 levels, with the most pronounced effects observed following treatment with 5 µM 18β-GA (Figure 2A).

Analysis of the p-AMPK/AMPK ratio did not reveal a significant increase following treatment, indicating that total AMPK and phosphorylated AMPK levels increased proportionally.

To investigate the contribution of AMPK signaling to the effects of 18β-GA, D283 cells were pre-treated with the AMPK inhibitor Compound C (10 µM). Pharmacological inhibition of AMPK significantly restored cell viability compared with cells treated with 18β-GA alone, suggesting the involvement of AMPK-dependent signaling in the anti-proliferative effects of 18β-GA (Figure 2B). Consistently, the increase in cleaved caspase-3 expression induced by 18β-GA was abrogated by Compound C (Figure 2C). Furthermore, Compound C reduced 18β-GA-induced AMPK expression and phosphorylation and restored NF-κB p65 expression levels (Figure 2D).

These results show that pharmacological inhibition of AMPK largely abrogated the cellular and molecular effects induced by 18β-GA, indicating that modulation of the AMPK/NF-κB axis contributes to the cytotoxic and pro-apoptotic effects of 18β-GA in D283 cells.

### 3.3. 18β-GA Reduces the Expression of SHH-Associated Transcriptional Markers in DAOY Cells

**Figure 3:**
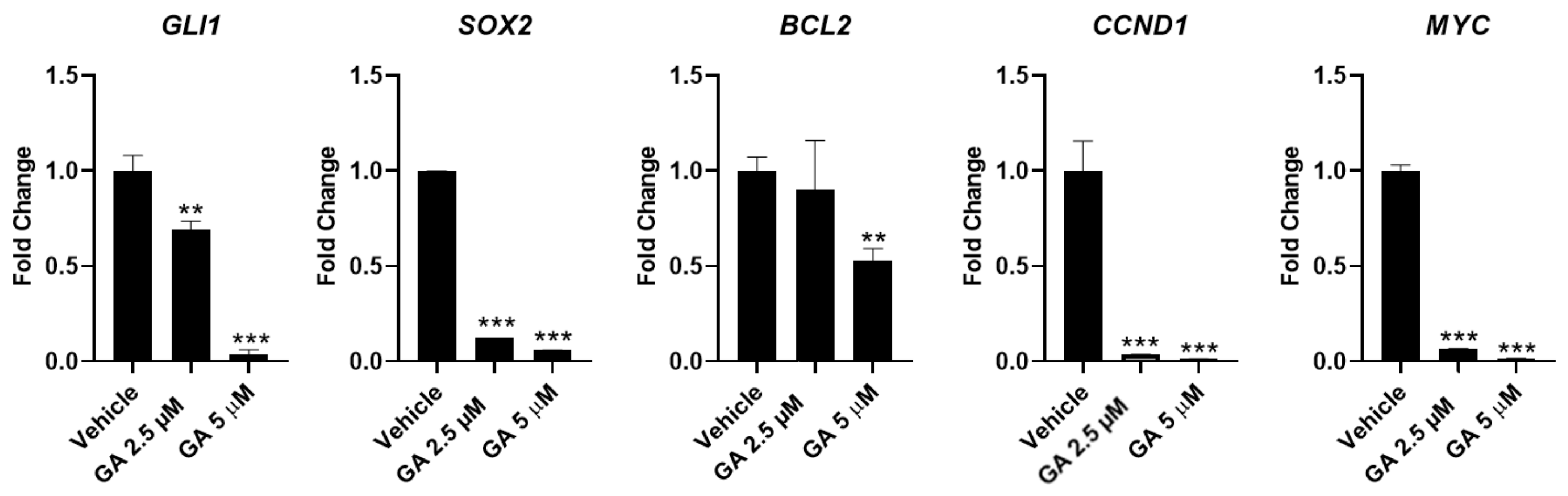
18β-GA modulates GLI1 and downstream SHH pathway gene expression in DAOY cells. DAOY cells were treated with 18β-GA (2.5 and 5 µM) for 24 h. Gene expression levels of *GLI1*, *SOX2*, *BCL2*, *CCND1*, and *MYC* were analyzed by RT-qPCR. Relative mRNA expression was calculated using the 2−ΔΔCt method and expressed as fold change relative to vehicle-treated controls. Data are presented as mean ± SD from three independent experiments (*n* = 3). Statistical significance was determined using one-way ANOVA followed by Tukey’s multiple-comparison post hoc test. ** *p* < 0.01; *** *p* < 0.001.

Since 18β-GA treatment significantly reduced DAOY cell viability without affecting AMPK expression and phosphorylation or NF-κB p 65 expression, we next investigated the expression of key components of the SHH signaling pathway by RT-qPCR to elucidate the molecular mechanisms underlying the effects of 18β-GA in SHH-associated MB cells. Treatment with 18β-GA resulted in a dose-dependent reduction in the expression of *GLI1* and its downstream target genes, including *SOX2*, *CCND1*, and *MYC* (Figure 3), well-known regulators of stemness, cell-cycle progression, and oncogenic signaling.

Instead, BCL2 was less responsive to treatment, showing a significant reduction only following exposure to 5 µM 18β-GA. In contrast, *GLI1*, *SOX2*, *CCND1*, and *MYC* expression levels were significantly decreased at both concentrations tested.

Collectively, these findings indicate that in DAOY cells, 18β-GA reduces the expression of SHH-associated transcriptional markers, supporting a subgroup-specific molecular response distinct from that observed in D283 cells.

### 3.4. 18β-GA Enhances Radiosensitivity in DAOY Cells

**Figure 4:**
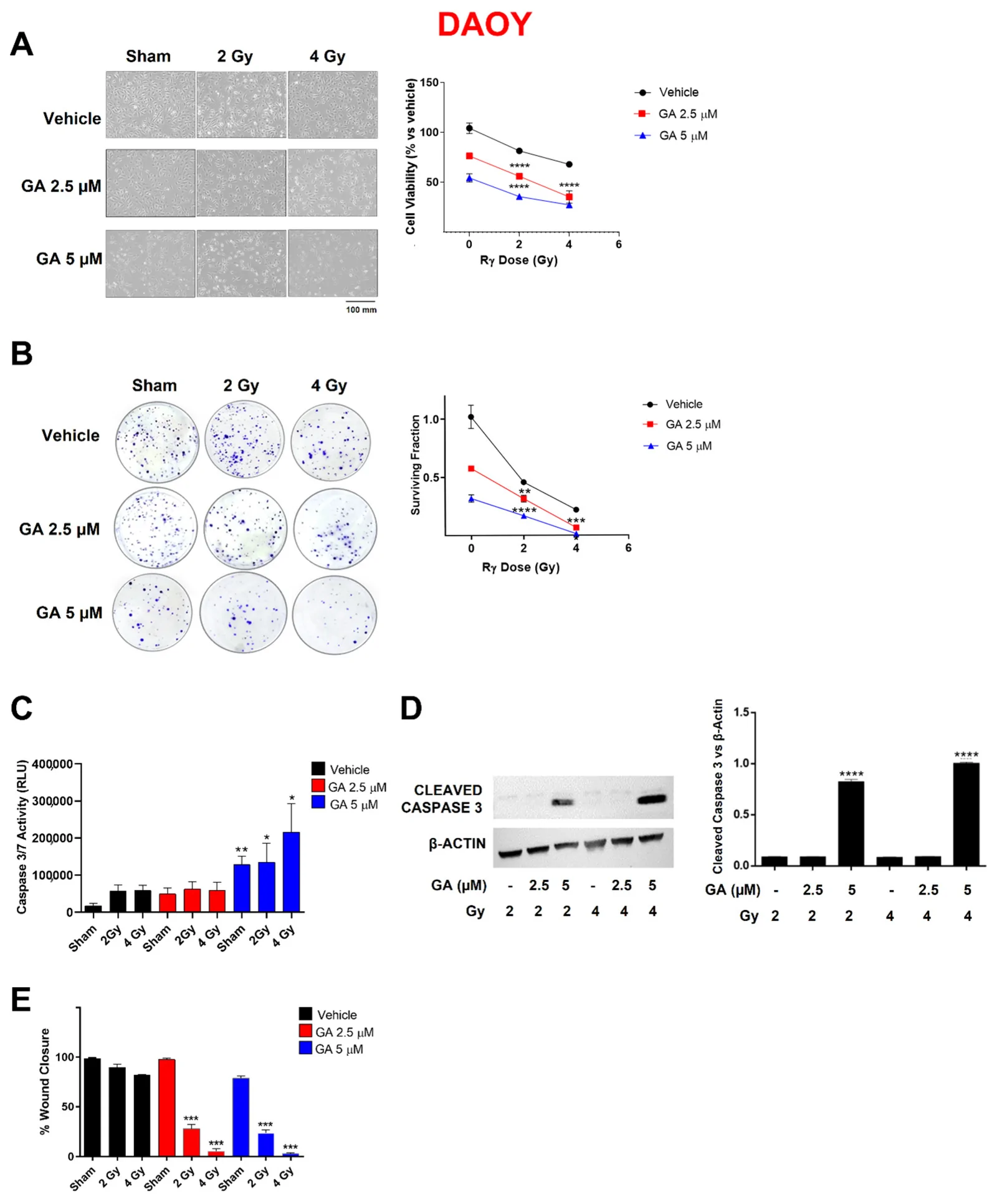
18β-GA enhances radiosensitivity and inhibits wound closure in DAOY cells. DAOY cells were pre-treated with 18β-GA (2.5 or 5 µM) for 24 h prior to γ-irradiation (2 or 4 Gy). Representative phase-contrast images (10× magnification, scale bar = 100 µm) and corresponding cell viability analysis are shown (**A**). Clonogenic survival was assessed by colony formation assay and expressed as surviving fraction (**B**). Caspase-3/7 activity was measured using the Caspa-se-Glo^®^ 3/7 Assay and expressed as relative luminescence units (RLU) (**C**). Representative immunoblots and densitometric analysis of cleaved caspase-3 expression are shown (**D**). The original uncropped Western blot images are provided in Supplementary Figure S3. Representative phase-contrast images of wound-healing assays (10× magnification, scale bar = 100 μm) are presented (**E**). Data are presented as mean ± SD from three independent experiments. Statistical significance was determined using two-way ANOVA followed by Tukey’s multiple-comparison post hoc test. * *p* < 0.05; ** *p* < 0.01; *** *p* < 0.001; **** *p* < 0.0001.

To evaluate the radiosensitizing potential of 18β-GA in SHH-associated MB cells, DAOY cells were pre-treated with 18β-GA (2.5 and 5 µM) for 24 h prior to exposure to γ-radiation. Cell viability analysis revealed that 18β-GA significantly enhanced radiation-induced cytotoxicity in DAOY cells (Figure 4A).

Whereas irradiation alone reduced cell viability to 81.6 ± 1.5% and 68.1 ± 2.6% following exposure to 2 and 4 Gy, respectively, pre-treatment with 18β-GA further decreased viability in a dose-dependent manner. At 2 Gy, cell viability declined to 56.0 ± 1.3% and 35.7 ± 2.7% in the presence of 2.5 and 5 µM 18β-GA, respectively. Similarly, following 4 Gy irradiation, viability was reduced to 35.4 ± 6.1% and 27.3 ± 2.7% after treatment with 2.5 and 5 µM 18β-GA, respectively. The lowest viability values were observed in cells receiving 5 µM 18β-GA in combination with irradiation. Notably, the combination of 5 µM 18β-GA with 2 Gy irradiation resulted in a degree of growth inhibition comparable to that observed with 4 Gy irradiation alone, supporting the ability of 18β-GA to enhance the cytotoxic efficacy of radiation treatment.

Clonogenic assays, the gold standard for assessing radiosensitivity, further confirmed the radiosensitizing activity of 18β-GA (Figure 4B). Treatment with 18β-GA alone reduced clonogenic survival in a dose-dependent manner. Irradiation alone decreased the surviving fraction to 0.46 ± 0.02 at 2 Gy and 0.22 ± 0.01 following exposure at 4 Gy, whereas pre-treatment with 5 µM 18β-GA further reduced it to 0.17 ± 0.01 and 0.013 ± 0.005, respectively. These values correspond to approximately 2.7- and 17-fold reductions relative to irradiation alone, indicating a marked impairment of long-term reproductive survival.

To account for the intrinsic anti-clonogenic effect of 18β-GA, surviving fractions were additionally normalized to the corresponding non-irradiated control at each drug concentration. Direct statistical comparisons of normalized surviving fractions revealed significantly lower clonogenic survival in cells receiving the combined treatment compared with radiation alone, further supporting the radiosensitizing effect of 18β-GA (Supplementary Table S2). In DAOY cells, normalization to the corresponding non-irradiated control revealed that 18β-GA did not further reduce clonogenic survival at 2 Gy, whereas a marked reduction was observed at 4 Gy. Consequently, the survival curves intersected within the investigated dose range, precluding a robust estimation of DEF_50_ (Supplementary Table S2).

Mechanistically, the reduced cell survival was associated with increased apoptotic signalling, particularly following combined treatment. Pre-treatment with 5 µM 18β-GA significantly increased both caspase-3/7 activity and cleaved caspase-3 levels compared with irradiation alone (Figure 4C,D).

Wound-healing assays further showed that irradiation alone had only a minimal effect on wound closure in DAOY cells, whereas the combined treatment produced a dose-dependent inhibition of wound closure comparable to that observed with 18β-GA alone (Supplementary Figure S2, Figure 4E).

Collectively, these findings indicate that 18β-GA enhances radiation-induced cytotoxicity, apoptosis, and loss of clonogenic potential in DAOY cells while maintaining reduced wound closure under combined-treatment conditions. The marked reduction in clonogenic survival following combined treatment further supports the radiosensitizing activity of 18β-GA in SHH-associated MB cells.

### 3.5. 18β-GA Potentiates Radiation Cytotoxicity in D283 Cells

**Figure 5:**
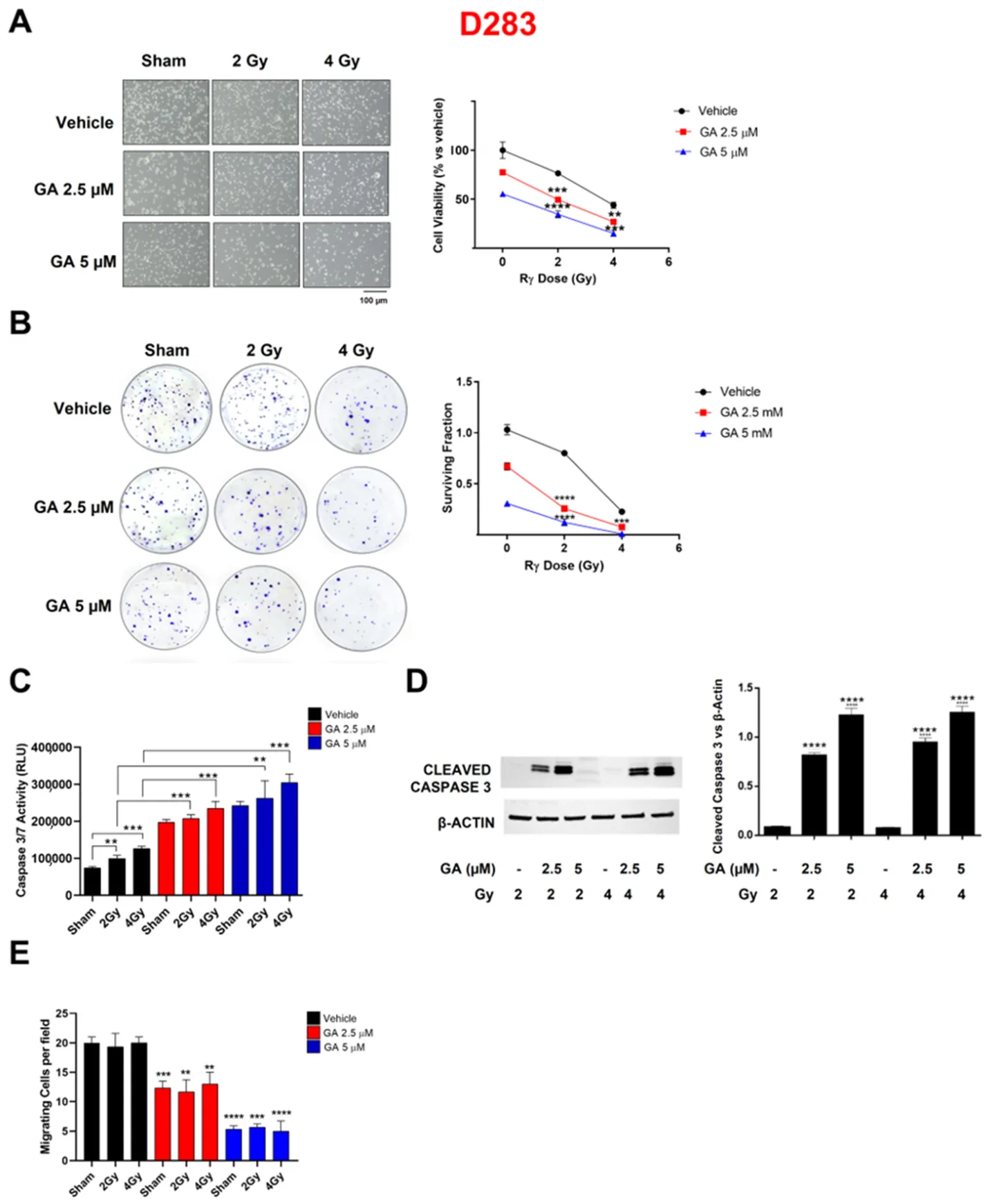
18β-GA potentiates radiation-induced cytotoxicity and reduces migratory capacity in D283 cells. D283 MB cells were pre-treated with 18β-GA (2.5 or 5 µM) for 24 h prior to γ-irradiation (2 or 4 Gy). Cell viability was evaluated 24 h after irradiation (**A**). Clonogenic survival was assessed by colony formation assay and expressed as surviving fraction (**B**). Caspase-3/7 activity was measured using the Caspase-Glo^®^ 3/7 Assay and expressed as relative luminescence units (RLU) (**C**). Representative immunoblots and densitometric analysis of cleaved caspase-3 expression are shown (**D**). The original uncropped Western blot images are provided in Supplementary Figure S3. Cell migration was evaluated using a Transwell assay and quantified as the mean number of migrated cells per field from five randomly selected microscopic fields per insert (**E**). Data are presented as mean ± SD from three independent experiments (*n* = 3). Statistical significance was determined using two-way ANOVA followed by Tukey’s multiple-comparison post hoc test. ** *p* < 0.01; *** *p* < 0.001; **** *p* < 0.0001.

To determine whether the radiosensitizing effects of 18β-GA observed in DAOY cells extended to non-SHH MB cells, cell viability, clonogenic survival, apoptosis, and migration were evaluated in D283 cells. Cells were pre-treated with 18β-GA (2.5 and 5 µM) for 24 h prior to exposure to γ-radiation. All endpoints were assessed 24 h after irradiation, with the exception of clonogenic survival, which was evaluated after 14 days. Cell viability assays confirmed the radiosensitizing effect of 18β-GA in D283 cells (Figure 5A).

Irradiation alone reduced viability in a dose-dependent manner (76.6 ± 2.4% at 2 Gy and 43.9 ± 3.1% at 4 Gy). Pre-treatment with 18β-GA significantly enhanced radiation-induced cytotoxicity. At 2 Gy, viability decreased to 49.6 ± 2.0% and 27.1 ± 2.9% in the presence of 2.5 and 5 µM 18β-GA, respectively. Similarly, following exposure to 4 Gy, viability was further reduced to 34.7 ± 3.6% and 15.2 ± 3.0%, respectively. The strongest effect was observed with 5 µM 18β-GA, which consistently yielded the lowest viability levels following irradiation, indicating a marked potentiation of radiation-induced cytotoxicity.

Notably, the reduction in cell viability observed following combined treatment was greater than that obtained with either treatment alone across all tested conditions. The combination of 5 µM 18β-GA and irradiation produced the most pronounced cytotoxic effect in D283 cells.

Clonogenic assays further supported both the anti-tumor and radiosensitizing activities of 18β-GA (Figure 5B). Treatment with 18β-GA alone substantially impaired the clonogenic potential of D283 cells, reducing the surviving fraction from 1.0 in vehicle-treated controls to approximately 0.67 and 0.31 following treatment with 2.5 and 5 µM 18β-GA, respectively. Irradiation alone induced a dose-dependent reduction in clonogenic survival, decreasing the surviving fraction to approximately 0.79 at 2 Gy and 0.22 at 4 Gy. The addition of 18β-GA markedly enhanced the effects of irradiation. Following exposure to 2 Gy, the surviving fraction decreased from 0.79 to 0.25 and 0.11 in the presence of 2.5 and 5 µM 18β-GA, respectively, corresponding to approximately 3-fold and 7-fold decreases in clonogenic survival relative to irradiated controls.

Similarly, following exposure to 4 Gy, pre-treatment with 2.5 µM 18β-GA reduced the surviving fraction to approximately 0.05, whereas treatment with 5 µM 18β-GA further reduced survival to approximately 0.01, corresponding to approximately 4-fold and greater than 20-fold reductions in clonogenic survival, respectively, compared with irradiation alone. Likewise, clonogenic survival was consistently lower in combined-treatment groups than in cells exposed to either 18β-GA or radiation alone, indicating a markedly greater suppression of long-term colony-forming capacity. To account for the intrinsic anti-clonogenic effect of 18β-GA, surviving fractions were additionally normalized to the corresponding non-irradiated control at each 18β-GA concentration. This complementary analysis yielded comparable results, confirming that clonogenic survival following irradiation remained lower in 18β-GA-treated cells than in vehicle-treated irradiated controls (Supplementary Table S2). Quantitative analysis of the normalized survival data yielded DEF_50_ values of 1.94 and 1.68 in cells treated with 2.5 and 5 µM 18β-GA, respectively, further supporting the radiosensitizing effect of 18β-GA (Supplementary Table S2). Together, these findings indicate a profound impairment of long-term clonogenic potential following combined treatment. The enhanced cytotoxic response was accompanied by increased apoptotic signaling. Whereas γ-irradiation alone induced caspase-3/7 activation without detectable cleaved caspase-3 expression, combined treatment with 18β-GA significantly increased both caspase-3/7 activity and cleaved caspase-3 levels, with stronger effects observed at 5 µM 18β-GA (Figure 5C,D).

Transwell migration assays showed that irradiation alone had only a minimal effect on D283 cell migratory capacity (Figure 5E). By contrast, combined treatment produced a dose-dependent reduction in cell migration comparable to that observed with 18β-GA alone, suggesting that the anti-migratory effect was primarily driven by 18β-GA.

Collectively, these findings demonstrate that 18β-GA exerts intrinsic anti-clonogenic activity and enhances radiation-induced cytotoxicity, apoptosis, and loss of clonogenic potential in D283 cells. Combined treatment reduced clonogenic survival by approximately 7-fold at 2 Gy and by more than 20-fold at 4 Gy relative to irradiation alone, supporting the radiosensitizing activity of 18β-GA in this non-SHH MB model.

## 4. Discussion

Radiotherapy remains central to MB treatment, but recurrence and treatment-related late effects continue to limit clinical outcomes, particularly in children and patients with high-risk disease. Craniospinal irradiation can produce substantial long-term neurocognitive and quality-of-life impairment, emphasizing the need for adjuvant agents that enhance tumor response without increasing radiation exposure [1,25].

Natural compounds are increasingly being investigated as radiosensitizers because they can simultaneously modulate oxidative stress, apoptosis, DNA damage responses, and pro-survival signaling. Phytochemicals such as curcumin, quercetin, and resveratrol have shown the potential to improve radiation response through these mechanisms [13,26]. Within this context, 18β-GA, a bioactive metabolite of *Glycyrrhiza glabra*, has demonstrated anti-proliferative, pro-apoptotic, and anti-migratory activity in non-small cell lung cancer, pituitary adenoma, renal cancer, and other experimental tumor models [27,28,29]. Our findings extend this spectrum to MB and indicate that 18β-GA exerts antitumor effects through model-dependent molecular responses that converge on increased apoptosis and radiation sensitivity.

In D283 cells, 18β-GA increased both total AMPK and phosphorylated AMPK protein levels and reduced NF-κB expression. Although the p-AMPK/AMPK ratio was not significantly altered, pharmacological inhibition experiments supported the involvement of the AMPK/NF-κB axis in the cellular response to 18β-GA. Pharmacological inhibition of AMPK attenuated 18β-GA-induced cytotoxicity and apoptosis, supporting a functional contribution of AMPK signaling to the response. This finding is consistent with evidence that AMPK can restrain MB growth and metastatic potential through modulation of NF-κB signaling [30]. Although previous studies were conducted mainly in SHH-driven models [31,32], our results suggest that modulation of the AMPK/NF-κB axis may also be relevant in Group 3/Group 4-associated cells.

The role of AMPK in MB nevertheless appears highly context-dependent. Chronic AMPK inactivation has also been reported to delay SHH-MB progression through suppression of mTORC1 signaling and depletion of tumor stem cells [32]. Moreover, AMPK activation may represent an adaptive response to metabolic or cellular stress rather than a tumor-suppressive mechanism per se [33]. Genetic modulation of AMPK will therefore be required to establish its direct causal role in the response to 18β-GA and to define how molecular background influences its biological effects.

DAOY cells displayed a distinct response, with limited modulation of the AMPK/NF-κB axis but marked reductions in the expression of SHH-associated transcriptional markers. 18β-GA reduced GLI1 expression together with the downstream targets SOX2, CCND1, and MYC, which regulate proliferation, stemness, and tumor progression. The reduction in SOX2 and MYC is particularly relevant because these factors have been associated with aggressive disease, treatment resistance, and unfavorable outcomes in MB [34,35]. Quiescent SOX2-positive populations have also been implicated in SHH MB maintenance and relapse [36,37]. Thus, reduced expression of SHH-associated transcriptional markers represents a plausible basis for the effects of 18β-GA in DAOY cells, although pathway-rescue experiments are required to establish causality.

18β-GA also induced apoptosis in DAOY cells, which harbor TP53 alterations. Given the association of TP53 abnormalities with aggressive and treatment-resistant SHH-MB [38], the persistence of an apoptotic response in this model is noteworthy. However, because the contribution of TP53 was not directly examined, p53-independent apoptosis cannot be inferred from the present data. Mitochondrial dysfunction and ROS-dependent apoptosis have been reported following 18β-GA treatment in other experimental systems [39,40], but their involvement in DAOY cells remains to be determined.

Beyond its cytotoxic activity, 18β-GA reduced wound closure in DAOY cells and migratory capacity in D283 cells. This effect may be biologically relevant because MB disseminates through cerebrospinal fluid pathways and frequently produces leptomeningeal metastases [41]. Anti-migratory effects of 18β-GA have previously been linked to oxidative stress, invasion-related signaling, and cytoskeletal regulation in lung and gastric cancer cells [16,17], while similar properties have been reported for other plant-derived compounds [42]. Although migration assays cannot reproduce the complexity of metastatic dissemination, and the reduction in wound closure observed in DAOY cells may also reflect effects on cell viability and proliferation, these findings support further investigation of 18β-GA in models that incorporate invasion, cerebrospinal fluid dissemination, and the tumor microenvironment.

A major translational finding of this study is the enhanced radiation response produced by 18β-GA in both MB models. Combined treatment reduced cell viability and, more importantly, markedly impaired clonogenic survival compared with irradiation alone. At 5 μM, 18β-GA reduced clonogenic survival in DAOY cells by approximately 2.7-fold after 2 Gy and 17-fold after 4 Gy. In D283 cells, the corresponding reductions were approximately sevenfold and more than twentyfold. These findings demonstrate a profound loss of long-term reproductive capacity and support the radiosensitizing potential of 18β-GA. Consistent with these findings, quantitative analysis of normalized clonogenic survival curves yielded DEF_50_ values of 1.94 and 1.68 in D283 cells treated with 2.5 and 5 μM 18β-GA, respectively. In DAOY cells, however, the survival curves intersected within the tested dose range, precluding robust estimation of DEF_50_ despite a clear reduction in clonogenic survival at 4 Gy.

Although the combination treatment consistently produced greater reductions in viability and clonogenic survival than either treatment alone, the present study was not designed to formally assess pharmacological synergy. Quantitative analyses, including combination index calculations, sensitizer enhancement ratio determination, and isobologram-based approaches, will be required in future studies to distinguish additive from synergistic interactions and to provide a more rigorous characterization of the observed treatment effects.

Radiosensitization occurred despite the distinct upstream responses of DAOY and D283 cells, suggesting convergence on common determinants of radiation survival. In both models, combined treatment increased caspase-3/7 activity and cleaved caspase-3 expression, indicating that enhanced apoptotic susceptibility contributes to the response. Apoptosis is unlikely to account fully for the marked reduction in clonogenic survival, however, and additional mechanisms may be involved.

Phytochemical radiosensitizers can enhance radiation response through ROS generation, inhibition of pro-survival signaling, and impairment of DNA damage repair [43,44]. The reduction in NF-κB p65 protein levels and the decreased expression of SHH-associated transcriptional markers observed here may contribute to reduced survival signaling following irradiation. Because radiation-induced clonogenic death is strongly influenced by the persistence of DNA double-strand breaks, future studies should determine whether 18β-GA alters DNA damage induction, repair kinetics, or repair fidelity. Analyses of γH2AX and 53BP1 foci, homologous recombination and non-homologous end-joining pathways, and residual genomic lesions would help define the mechanisms underlying the radiosensitizing phenotype.

Notably, 5 μM 18β-GA combined with 2 Gy produced effects on viability and clonogenic survival comparable to those observed with higher radiation doses alone. This result raises the possibility that 18β-GA could contribute to radiation dose-de-escalation strategies. Such an application would be particularly relevant in pediatric neuro-oncology, where limiting radiation-associated late toxicity is a major therapeutic objective. Nevertheless, equivalence of biological effects in vitro does not demonstrate equivalent tumor control. Complete radiation survival curves, linear-quadratic modeling, and sensitizer enhancement ratio calculations are required before conclusions regarding dose reduction can be drawn.

18β-GA has also shown antioxidant and cytoprotective activity in non-malignant tissues [45], including attenuation of radiation-induced skin injury through Nrf2/HO-1 activation [18]. Similar context-dependent radiosensitizing and radioprotective effects have been described for curcumin and quercetin [46,47]. These observations raise the possibility of a favorable differential effect in which 18β-GA sensitizes malignant cells while protecting normal tissues. However, this remains speculative and requires direct comparison of tumor and non-malignant neural cells under equivalent irradiation and treatment conditions to establish tissue selectivity and define the therapeutic window of 18β-GA. Taken together, our findings indicate that 18β-GA exerts antitumor effects through distinct molecular mechanisms depending on the MB cellular context. In D283 cells, the response is associated with modulation of the AMPK/NF-κB axis, whereas in DAOY cells it is associated with reduced expression of SHH-associated transcriptional markers. Despite these differences, both models converge on increased apoptosis, reduced clonogenic survival, and enhanced radiosensitivity, supporting a multifaceted mechanism of action for 18β-GA in MB.

Several limitations should be acknowledged. The study relied on established cell lines that do not reproduce the molecular heterogeneity, microenvironment, or treatment response of primary MB. DAOY cells represent only part of the SHH disease spectrum, while the classification of D283 cells as a Group 3/Group 4 model remains imperfect. Importantly, the absence of patient-derived and in vivo validation limits the translational interpretation of the present findings, as the therapeutic efficacy of 18β-GA may be influenced by tumor heterogeneity, blood–brain barrier penetration, pharmacokinetics, and interactions with the tumor microenvironment. Although DEF_50_ values could be estimated in D283 cells, the limited number of radiation dose points and the absence of complete survival curves prevented a comprehensive radiobiological characterization. In DAOY cells, intersecting survival curves precluded robust DEF_50_ estimation.

Moreover, the pharmacokinetics, safety, blood–brain barrier penetration, and in vivo efficacy of 18β-GA remain unknown in MB. In addition, the antitumor and radiosensitizing effects described here were observed at micromolar concentrations in vitro, which may not directly correspond to concentrations achievable and sustainable within human tumor tissues. Therefore, further studies addressing pharmacokinetics, bioavailability, formulation strategies, tissue distribution, and blood–brain barrier penetration will be required to determine whether therapeutically effective concentrations can be achieved and maintained within tumor tissue. Although nanoparticle and nose-to-brain delivery systems may improve central nervous system exposure [48], their suitability for tumor-directed delivery requires experimental validation.

## 5. Conclusions

This study identifies 18β-GA as an antitumor agent capable of markedly enhancing radiation response in biologically distinct MB models. Its effects involve model-dependent modulation of the AMPK/NF-κB axis and reduced expression of SHH-associated transcriptional markers but converge on increased apoptotic susceptibility and loss of clonogenic survival. This convergence across distinct molecular backgrounds represents the principal mechanistic and translational advance of the study. Validation in patient-derived models and in vivo systems, together with quantitative radiobiological and DNA-repair analyses, as well as studies addressing pharmacokinetics, blood–brain barrier penetration, and drug delivery strategies, will be essential to determine whether 18β-GA can be developed as a clinically meaningful radiotherapy adjuvant and ultimately contribute to more effective, less toxic treatment strategies for pediatric MB.

## Data Availability

The datasets generated and/or analysed during the current study are available from the corresponding author on reasonable request.

## Supplementary Materials

The following supporting information can be downloaded at: https://www.mdpi.com/article/10.3390/cancers18193233/s1, Figure S1: AMPK, p-AMPK, and NF-κB expression in DAOY cells following 18β-GA treatment. Figure S2: Representative wound-healing assay images of DAOY MB cells co-treated with 18β-GA and γ-radiation; Figure S3: The original uncropped Western blot images; Table S1: Primer sequences used for RT qPCR; Table S2: Clonogenic survival and quantitative assessment of radiosensitization by 18β-glycyrrhetinic acid (18β-GA) in DAOY and D283 medulloblastoma cells.

## Abbreviations

The following abbreviations are used in this manuscript:

18β-GA	18β-glycyrrhetinic acid
AMPK	AMP-activated protein kinase
BCL2	B-cell lymphoma 2
DAOY	Medulloblastoma cell line (SHH subgroup)
D283	Medulloblastoma cell line (Group 3/4)
GLI1	GLI family zinc finger 1
NF-κB	Nuclear factor kappa-light-chain-enhancer of activated B cells
p-AMPK	Phosphorylated AMP-activated protein kinase
qPCR	Quantitative polymerase chain reaction
ROS	Reactive oxygen species
SHH	Sonic Hedgehog signaling pathway
SOX2	SRY-box transcription factor 2
TP53	Tumor protein p53
MYC	MYC proto-oncogene
