# Supplementary material for: 18β-Glycyrrhetinic Acid Exerts Subgroup-Specific Antitumor Activity and Enhances Radiosensitivity in Medulloblastoma
Source: Cancers (Basel). 2026 Oct 8;18(19):3233. doi: 10.3390/cancers18193233 (PMC13664084; doi:10.3390/cancers18193233)
Supplement: Supplementary file 1 [file cancers-18-03233-s001.zip › cancers-4583346-supplementary.pdf]

cancers

MDPI

DAOY

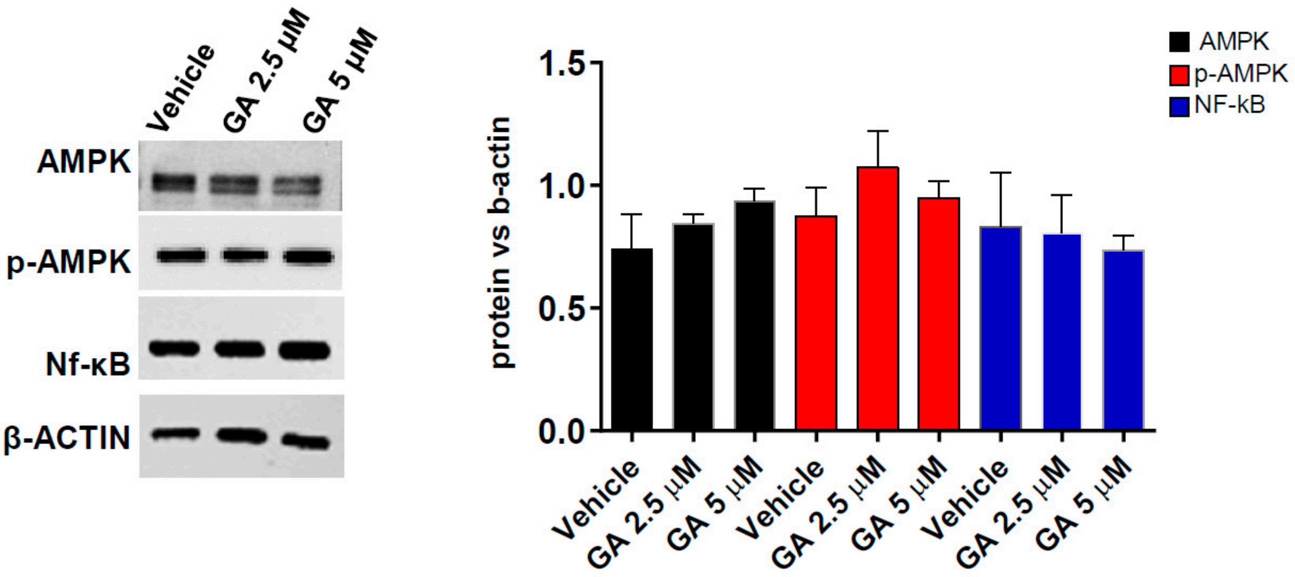

Figure S1. AMPK, p-AMPK, and NF-κB expression in DAOY cells following 18β-GA treatment. Representative immunoblot images showing total AMPK, phosphorylated AMPK (p-AMPK), NF-κB, and β-actin (loading control) expression in DAOY cells treated with vehicle or 18β-GA (2.5 and 5 µM) for 24 h. Corresponding densitometric analyses were normalized to β-actin and are presented as mean ± SD from three independent experiments. Statistical comparisons between vehicle-treated cells and each 18β-GA treatment group were performed using an unpaired Student's t-test with Welch's correction. No significant differences were observed for any protein analyzed (n.s., $p > 0.05$).

DAOY

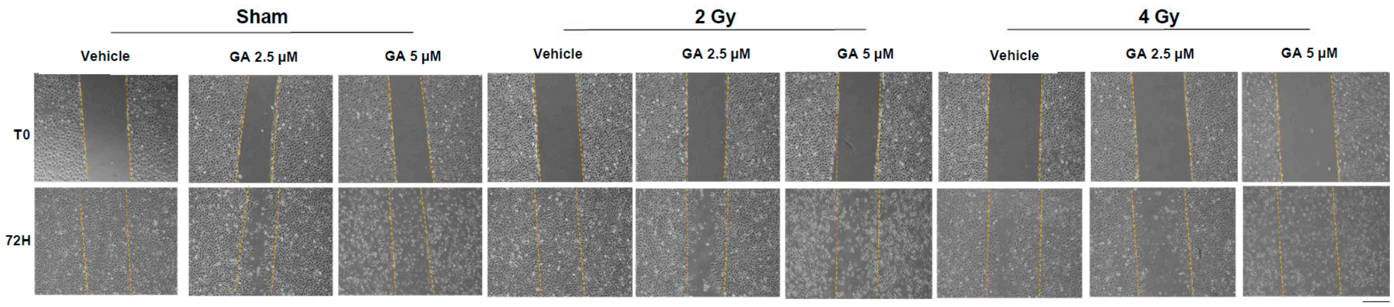

 https://doi.org/10.3390/cancers18193233

Figure S2. Representativewound-healingassayimagesofDAOYMBcellsco-treatedwith18β-GAandγ-radiation. DAOYcellswerepre-treatedwith18β-GA(2.5and5μM)for24hpriortoγ-irradiation(2and4Gy).Representativephase-contrastimages(10×magnification,scalebar=100μm)werecapturedatthetimeofscratchgeneration(0h,upperpanels)and72hlater(lowerpanels).Dashedyellowlinesdelineatethewoundarea.

Figure 1E, F

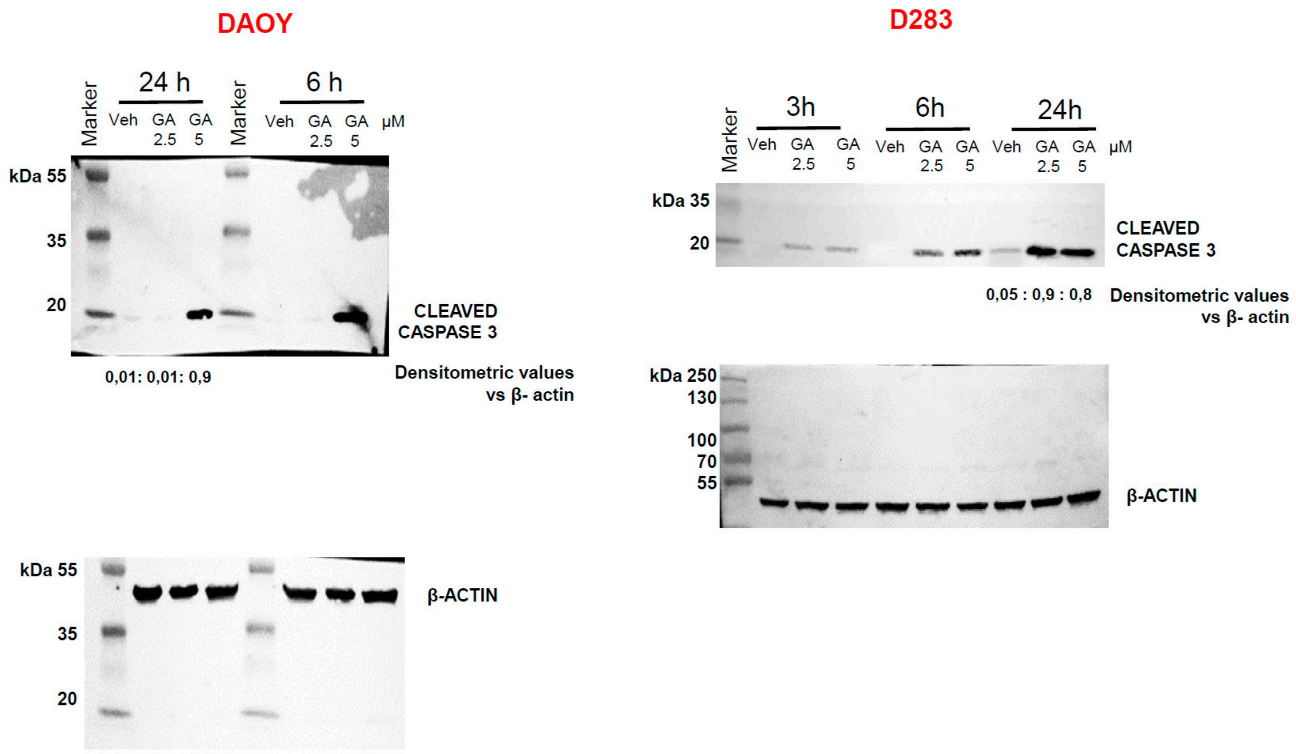

Figure 2

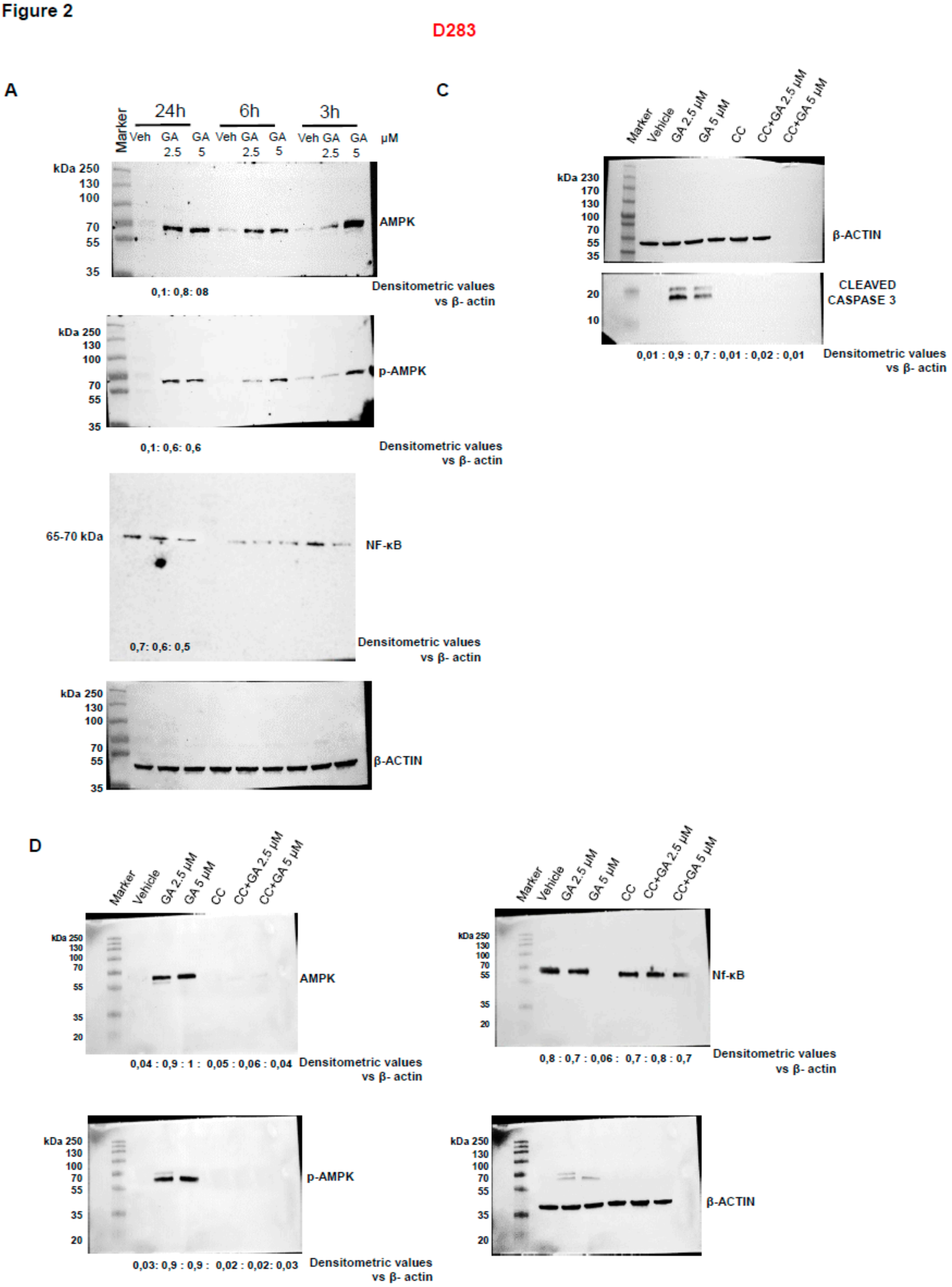
D283
A
Marker
24h
6h
3h
Veh
GA 2.5
GA 5
µM
kDa 250
130
100
70
55
35
AMPK
0,1: 0,8: 08
Densitometric values vs β- actin
p-AMPK
0,1: 0,6: 0,6
Densitometric values vs β- actin
65-70 kDa
NF-κB
0,7: 0,6: 0,5
Densitometric values vs β- actin
β-ACTIN
C
Marker
Vehicle
GA 2.5 µM
GA 5 µM
CC
CC+GA 2.5 µM
CC+GA 5 µM
kDa 230
170
β-ACTIN
CLEAVED CASPASE 3
20
10
0,01 : 0,9 : 0,7 : 0,01 : 0,02 : 0,01
Densitometric values vs β- actin
D
AMPK
0,04 : 0,9 : 1 : 0,05 : 0,06 : 0,04
Densitometric values vs β- actin
Nf-κB
0,8 : 0,7 : 0,06 : 0,7 : 0,8 : 0,7
Densitometric values vs β- actin
p-AMPK
0,03: 0,9 : 0,9 : 0,02 : 0,02: 0,03
Densitometric values vs β- actin
β-ACTIN

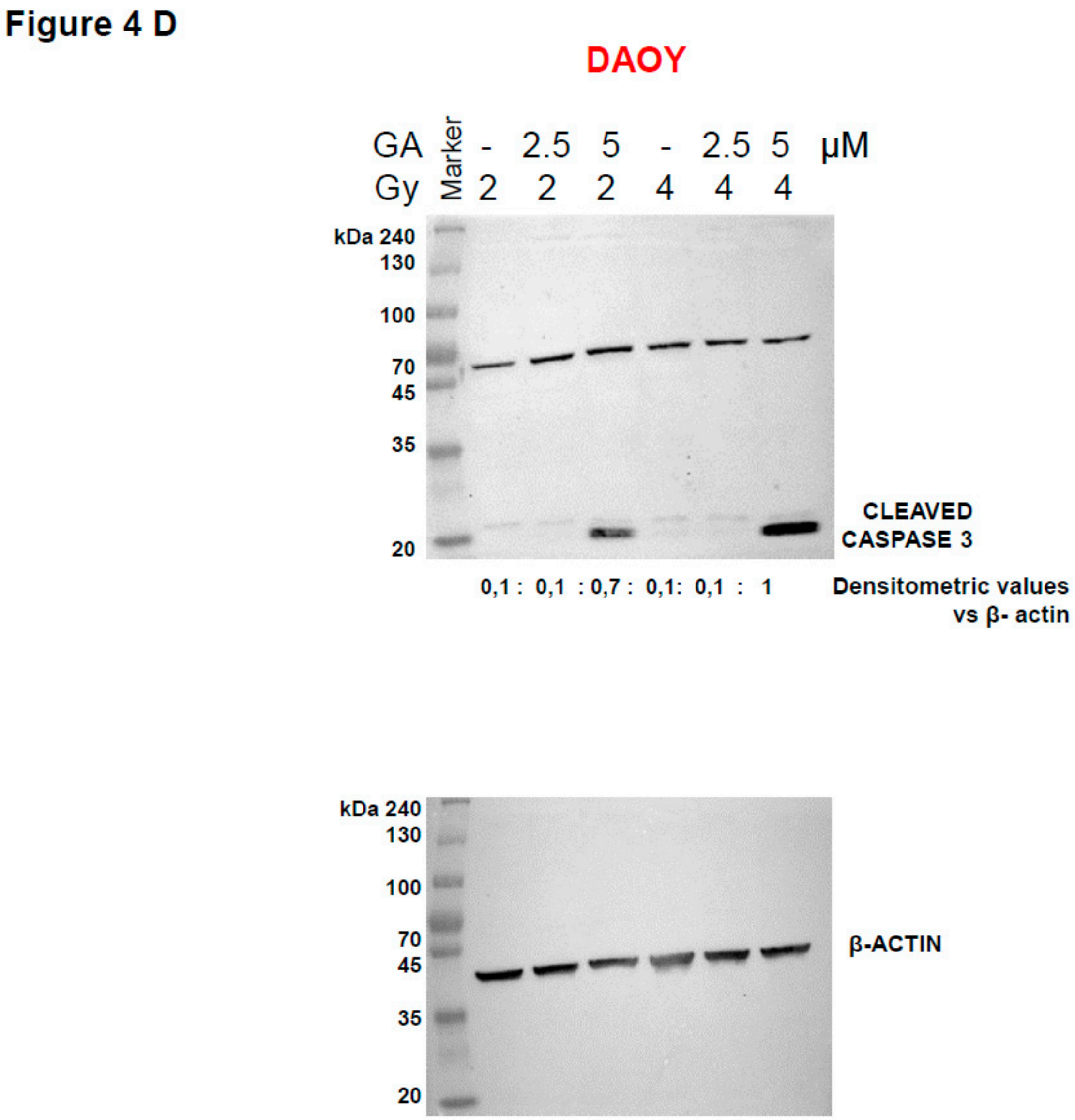
Figure 4 D
DAOY
GA - 2.5 5 - 2.5 5 μM
Gy 2 2 2 4 4 4
Marker
kDa 240
130
100
70
45
35
20
CLEAVED CASPASE 3
0,1 : 0,1 : 0,7 : 0,1: 0,1 : 1
Densitometric values vs β- actin
kDa 240
130
100
70
45
35
20
β-ACTIN

Figure 5 D

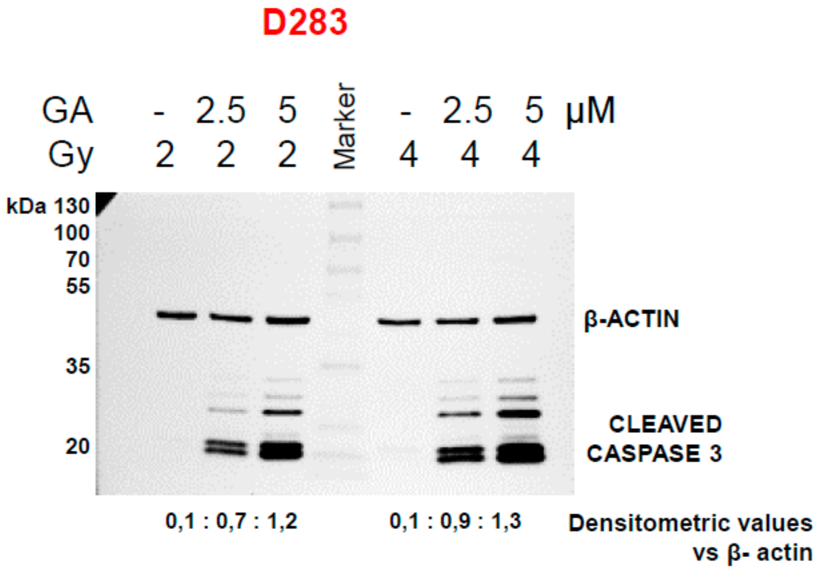
D283
GA - 2.5 5 Marker - 2.5 5 µM
Gy 2 2 2 4 4 4
kDa 130
100
70
55
35
20
β-ACTIN
CLEAVED CASPASE 3
0,1 : 0,7 : 1,2
0,1 : 0,9 : 1,3
Densitometric values vs β- actin

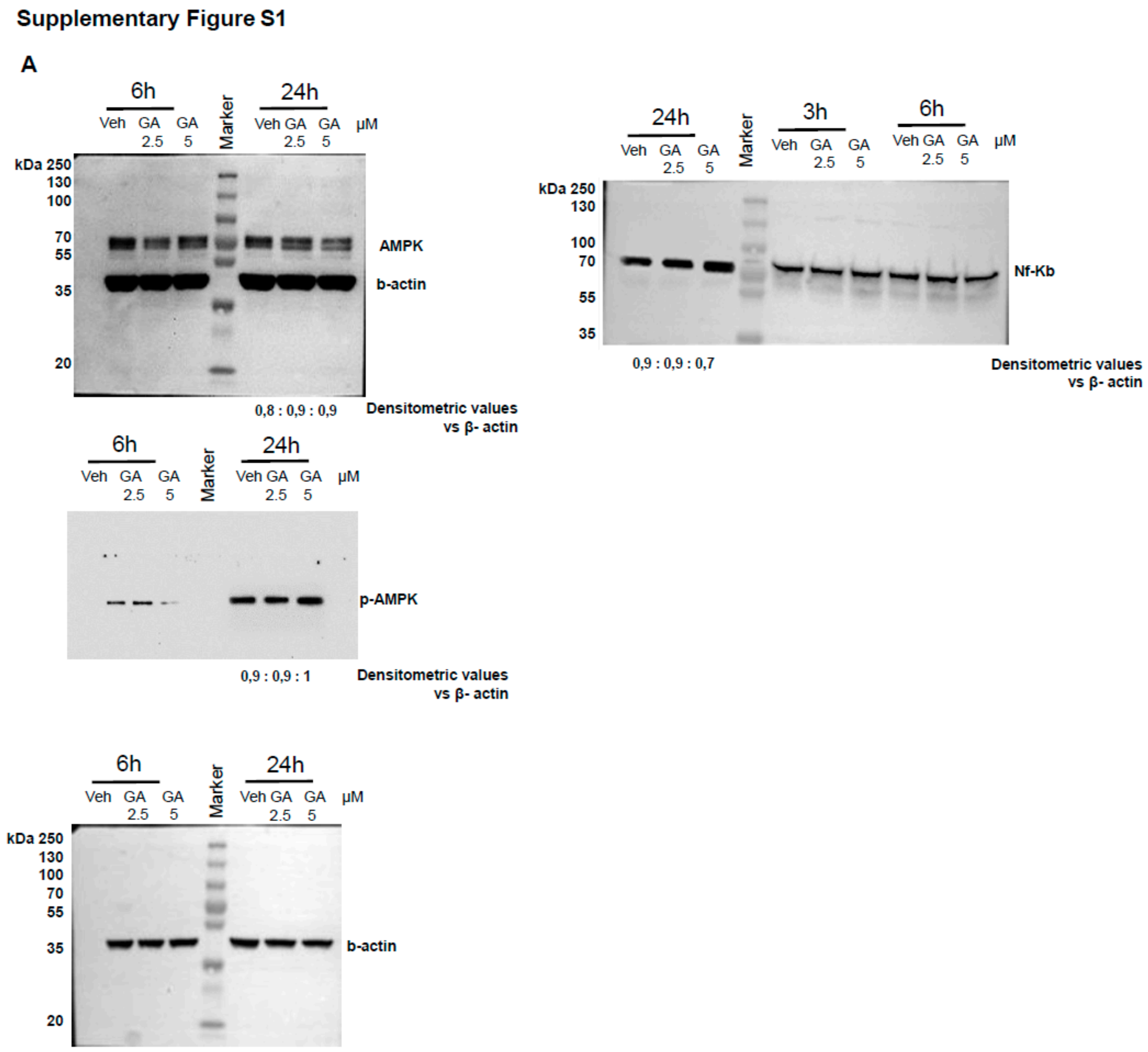

Figure S3. Original Uncropped Western Blot images. Representative original, uncropped Western blot scans for the experiments shown in Figures 1E,F, 2A,C,D, 4D, 5D and Supplementary Figure S1. Protein markers and densitometric values normalized to β-actin are indicated.

Table S1. Primer sequences used for RT qPCR.

Gene	Forward primer (5'-3')	Reverse primer (5'-3')
GLI1	GAATGCTGCCATGATGCTA	ACCATGGAGGTCCCAACTT
SOX2	GAGCTTTGCAGGAAGTTTG	GCAAGAAGCCTCTCCTTGAA
BCL2	TCGCCCTGGGATGCTGA	CAGAGACAGCCAGGAGAAATCA
CCND1	TGTGCATCTACACCGACAACT	CACAGAGGGCAACGAAGGT
MYC	CCTGGTGCTCCATGAGGAGAC	CAGACTCTGACCTTTTGCCAGG
GAPDH	GCACCGTCAAGGGTGAGAAC	GAGGGATCTCGCTCCTGGA

Table S2. Clonogenic survival and quantitative assessment of radiosensitization by 18β-glycyrrhetinic acid (18β-GA) in DAOY and D283 medulloblastoma cells. Surviving fractions (SF) were normalized, within each independent experiment, to the corresponding non-irradiated control

treated with the same concentration of 18β-GA (SF = 1). Data are presented as mean ± SD from three independent biological experiments. Dose enhancement factors at 50% survival (DEF_{50}) were calculated as the ratio between the radiation dose required to achieve SF = 0.50 in untreated cells and that required in 18β-GA-treated cells, based on linear–quadratic fitting. In DAOY cells, DEF_{50} was not considered reliably estimable because the survival curves intersected within the experimentally tested dose range.

Cell line	18β-GA (μM)	0 Gy	2 Gy	4 Gy	Def_{50}
DAOY	0	1.00	0.448 ± 0.042	0.215 ± 0.029	-
	2.5	1.00	0.547 ± 0.030 *	0.114 ± 0.017 *	n.a.
	5	1.00	0.533 ± 0.068	0.040 ± 0.016 **	n.a.
D283	0	1.00	0.778 ± 0.033	0.220 ± 0.017	-
	2.5	1.00	0.383 ± 0.031 ***	0.114 ± 0.027 **	1.94
	5	1.00	0.401 ± 0.091 *	0.032 ± 0.031 **	1.68

Statistical significance was assessed using two-way ANOVA followed by Tukey's multiple-comparison test. *$p < 0.05$, **$p < 0.01$, ***$p < 0.001$. * n.a., not applicable/reliably estimable: in DAOY cells, survival curves intersect within the tested dose range, preventing a meaningful estimation of DEF_{50} from the available 0–4 Gy dataset.
